# Cytoplasmic glycoengineering of Apx toxin fragments in the development of *Actinobacillus pleuropneumoniae* glycoconjugate vaccines

**DOI:** 10.1186/s12917-018-1751-2

**Published:** 2019-01-03

**Authors:** Ian J. Passmore, Anna Andrejeva, Brendan W. Wren, Jon Cuccui

**Affiliations:** 1 Department of Pathogen Molecular Biology, London School of Hygiene and Tropical Medicine, Keppel Street, London, WC1E 7HT UK; 2 Cambridge Centre for Proteomics, Department of Biochemistry, University of Cambridge, Tennis Court Road, Cambridge, CB2 1QR UK

**Keywords:** N-linked glycosylation, *Actinobacillus pleuropnuemoniae*, Glycoengineering

## Abstract

**Background:**

*Actinobacillus pleuropneumoniae* is the causative agent of porcine pleuropneumonia and represents a major burden to the livestock industry. Virulence can largely be attributed to the secretion of a series of haemolytic toxins, which are highly immunogenic. *A. pleuropneumoniae* also encodes a cytoplasmic *N*-glycosylation system, which involves the modification of high molecular weight adhesins with glucose residues. Central to this process is the soluble *N*-glycosyl transferase, *ngt,* which is encoded in an operon with a subsequent glycosyl transferase, *agt*. Plasmid-borne recombinant expression of these genes in *E. coli* results in the production of a glucose polymer on peptides containing the appropriate acceptor sequon, NX(S/T). However to date, there is little evidence to suggest that such a glucose polymer is formed on its target peptides in *A. pleuropneumoniae.* Both the toxins and glycosylation system represent potential targets for the basis of a vaccine against *A. pleuropneumoniae* infection.

**Results:**

In this study, we developed cytoplasmic glycoengineering to construct glycoconjugate vaccine candidates composed of soluble toxin fragments modified by glucose. We transferred *ngt* and *agt* to the chromosome of *Escherichia coli* in order to generate a native-like operon for glycoengineering. A single chromosomal copy of *ngt* and *agt* resulted in the glucosylation of toxin fragments by a short glycan, rather than a polymer.

**Conclusions:**

A vaccine candidate that combines toxin fragment with a conserved glycan offers a novel approach to generating epitopes important for both colonisation and disease progression.

**Electronic supplementary material:**

The online version of this article (10.1186/s12917-018-1751-2) contains supplementary material, which is available to authorized users.

## Background

*Actinobacillus pleuropneumoniae* is the causative agent of porcine pleuropneumonia, which represents a significant burden to the pig industry worldwide. *A. pleuropneumonia* infections are highly contagious, often fatal and are characterised by necrotic and haemorrhagic lung legions. Sixteen serotypes have been implicated in causing disease, classified based on their capsular polysaccharide composition [1–3]. *A. pleuropneumoniae* virulence is largely attributed to the secretion of proteins ApxI, ApxII, ApxIII and ApxIV, which are members of the RTX family of pore-forming toxins [4]. Each serotype encodes and secretes each toxin in various combinations. While all serotypes are capable of causing disease, those that express ApxI and ApxII (1, 5, 9, 11, and) display greater virulence [4, 5]. No serotype identified to date encodes all 4 toxins.

Apx toxins are highly immunogenic and animals infected with *A. pleuropneumoniae* induce a strong production of reactive antibodies [6]. Consequently, the toxins represent the best candidates for effective vaccines against infection. The importance of Apx toxins as vaccine candidates has been demonstrated in a number of studies [7–12]. Currently, licensed vaccines are a mixture of these toxins (inactivated), combined with a 42 kDa outer membrane protein [13, 14]. These vaccines have demonstrated impressive efficacy, however, manufacture requires isolation and detoxification of ApxI, ApxII and ApxIII as well as the outer membrane protein, all from *A. pleuropneumoniae*. Therefore, an alternative, lower cost, production method that could improve vaccine efficacy is desired.

A number of *A. pleuropnuemoniae* surface structures have been reported to be involved in adhesion and colonisation including fimbriae [15], lipopolysaccharide (LPS) [16] and autotransporter adhesins [17]. Recently, a family of *N*-linked protein glycosylation pathways has been described. In the prototype pathway, characterised in *Haemophilus influenzae,* asparagine residues of the outer membrane adhesin protein, HMW1A, are decorated with hexose and dihexose residues by the cytoplasmic glycosyltransferase HMW1C [18, 19]. The genes encoding this system are collocated on the chromosome. Glycosylation of HMW1A by HMW1C has been shown to confer protection against proteolytic degradation during secretion and facilitates tethering of the adhesin to the cell surface [18]. An analogous adhesin/glycosyl transferase system has also been identified in *A. pleuropneumoniae,* the in vitro and in vivo properties of which have been recently reported [20–23]. Unlike *hmw1A/C,* the *A. pleuropneumoniae N*-glycosyl transferase gene, *ngt*, is not located adjacent to an obvious acceptor protein adhesin gene. This is similar to the recently described systems of *Kingella kingae* and *Aggregatibacter aphrophilus* [24], whereby these ‘orphan’ HMWC family of transferases are distally encoded from their targets.

Unique to *A. pleuropneumoniae*, the *ngt* operon also encodes a polymerising α1,6-glucosyltransferase, *agt,* which elongates *N*-linked glucose. To date, confirmed substrates of this system are limited to autotransporter adhesins [21]. NGT is an inverting glycosyl transferase that exhibits similar acceptor site specificity to the eukaryotic oligosaccharyltransferase consensus sequon, N*X*(S/T), where X is any amino acid except proline [20]. Recombinant expression of *ngt* and *agt* with an appropriate acceptor protein results in the modification of target asparagine with a glucose homopolymer with a chain of up to 29 repeat units [23]. This modification is detectable by immunoblot analysis with an antibody raised against a tetrasaccharide of α1,6-linked glucose (dextran). However, immunofluorescence studies failed to detect this epitope in permeabilised *A. pleuropneumoniae* cell extracts. This suggests that although *ngt* and *agt* have the capacity to form extended glucose polymer, this is not observed in native-like conditions, indicating that the native polymer may not exceed 4 units. Deletion of *ngt* and *agt* was shown to reduce *A. pleuropneumoniae* adhesion to a human epithelial cell line [23], demonstrating a potential importance in colonisation and virulence. Furthermore, the glycosylation system is absolutely conserved across *A. pleuropnuemoniae* isolates, making it one of the most attractive targets for subunit vaccine development [25].

Glycoconjugate vaccines provide superior immunity compared with polysaccharides alone. The attachment of a sugar molecule to a typical T cell antigen (such as protein) induces memory T proliferation, memory B cell development and formation of polysaccharide-specific IgM to IgG switching. The *H. influenzae* HMW1/2 glycosylated adhesins have been extensively studied as potential vaccine candidates against otitis media infection in children and chronic obstructive pulmonary disease in adults due to their well-conserved nature (encoded in 75% *H. influenzae* strains), abundance of epitopes on the cell surface and ability to induce a protective immune response [26–29]. As such, a vaccine that combines the polyglucose glycan (highly conserved, abundant on the cell surface) and the Apx toxins (induces a protective immune response) has the potential to confer enhanced protection against all *A. pleuropneumoniae* serotypes.

Recombinant expression of these components in *E. coli* represents a convenient way to produce an inexhaustible and purified supply of glycoconjugate-based vaccines, thereby reducing production costs compared with current methods. However, the use of biological conjugation systems to functionally transfer key glycosyl transferase genesto the chromosome of a convenient host organism has the potential to reduce costs further still. Stable integration of genes coding for transferase enzymes has a number of advantages over current methods for recombinant expression of vaccine candidates. Firstly, it reduces the metabolic burden of maintaining energetically costly and often unstable plasmids, which can result in reduced cell viability and reduced yield in batch fermentation [30]. Any move to reduce this burden should, in theory, contribute to improving the overall yield and reduce cost of production. A second advantage of mastering this method is that it unlocks the potential to generate live attenuated vaccine strains expressing novel glycan antigens. Stable expression of a transferase system (with or without a corresponding acceptor protein) in a clinically relevant attenuated pathogen would confer protection against the pathogen itself and against the resulting glycan introduced from a second pathogen. The NGT system of *A. pleuropneumoniae* lends itself well to this concept, as it requires a relatively short acceptor sequon and the system is comprised of only two transferases. Although NGT does display some substrate preference to autotransporter adhesins, it can glucosylate other protein classes that contain the correct acceptor sequon [21]. Introduction of NGT/AGT alone should result in the formation of a cloned supply of host peptides modified by *A. plueropneumoniae* glycan.

We describe a method for the construction of candidate glycoconjugate vaccines against *A. pleuropneumoniae*. We use transposon-based integration system to generate a recombinant *E. coli* cell with a single chromosomally encoded copy of *ngt* and *agt*, which we use to modify soluble fragments of the immunogenic Apx toxins. Our results suggest that modification of Apx toxins by this system results in a glycoconjugate with a native-like glucose modification.

## Results

### Chromosomal insertion of *ngt* and *agt*

**Fig. 1:**
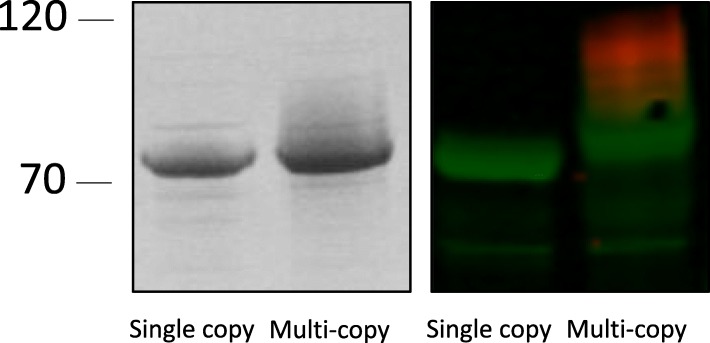
Demonstration of glycosylation of the peptide AtaC_1866–2428_ by a single chromosomal copy or multiple plasmid copies of the *ngt* and *agt* operon. Glycosylated AtaC was resolved by SDS-PAGE and detected by coomassie staining (left panel) or immunoblot analysis (right panel), probed with anti-6xHis (green) and anti-dextran (red) antibody

One of the key considerations for the construction of suitable *E. coli* strains for glycoengineering is the organisation and fine-tuned expression of acceptor proteins, glycosyl transferases and sugar biosynthesis loci onto compatible expression plasmids. Finding plasmids with appropriate compatibility groups can be a limiting factor in the engineering process and antibiotic-based maintenance of plasmids can impede cell growth. One way to obviate the use of multiple plasmids is the integration of one or more of these components onto the *E. coli* chromosome. Furthermore, artificially increased expression of recombinant transferases can result in artefactual products. For example, *ngt* and *agt* expression from a high-copy plasmid (ColE1 ori, 15–20 copies) resulted in hyper-glycosylation of the *A. pleuropneumoniae* autotransporter adhesin fragment, AtaC_1866–2428_. Figure 1, (lanes 2 and 4) demonstrates the presence of a high molecular weight smear visible above the point at which AtaC should migrate when resolved by SDS-PAGE. NGT decorates AtaC_1866–2428_ with hexose at 15 Asn sites with up to 29 units at each site [23]. However, immunoreactivity with anti-dextran monoclonal antibody was not observed in *A. pleuropneumoniae* cell extracts and only single hexose units on autotransporter adhesins were detected by mass spectrometry analysis [21]. Thus, we concluded that ectopic expression of *ngt* and *agt* from high copy number plasmids results in an artificial, hyper-polymerised glycan on its target acceptor protein.

To overcome these two constraints we used the pUT mini-Tn*5* transposon [31] to integrate *ngt* and *agt*, under the transcriptional control of a *lac* promoter, into the chromosome of *E. coli* BL21 cells. Our transposon-delivered cargo also included the *lacI* repressor. This generated an *E. coli* strain with a single chromosomal copy of *ngt* and *agt*, transcriptional controlled by *lacI*/T7 polymerase for inducible protein expression. Glycosylation of AtaC using this strain resulted in the absence of a smear on a gel, suggesting that this hyperglycosylation was indeed a product of multicopy plasmid-borne transferase expression.

### ApxIA calcium-binding domain is a soluble suitable candidate for glycoengineering

**Fig. 2:**
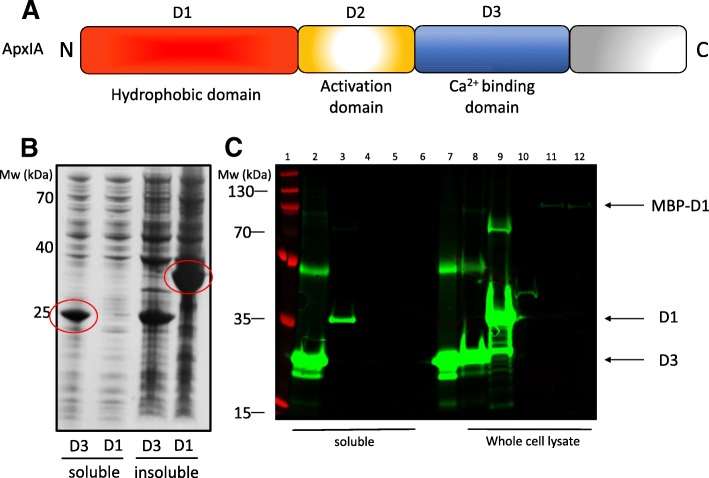
Expression and solubility of ApxIA peptide fragments. **a**- Schematic representation of the domain architecture of ApxIA. **b**- The ‘hydrophobic’ domain (D1) and Ca^2+^-binding domain (D3) of ApxIA were recombinantly expressed in *E. coli*, lysed and soluble and insoluble fractions were resolved by SDS-PAGE. **c**- Expression and solubility of ApxIA fragments immunoblot detected using an anti-6xHis (green) antibody. Lane 1: Ladder; lane 2: *E. coli* expressing ApxIAD3 (soluble); lane 3: ApxIAD1; lane 4: ApxIAD2; lane 5: MBP-ApxIAD1 fusion; lane 6: MBP_Nlinker_-ApxIAD1 fusion; lane 7: ApxIAD3 Ni-NTA affinity purified; lane 8 ApxIAD3 (whole cell lysate); lane 9: ApxIAD1; lane 10: ApxIAD2; lane 11: MBP-ApxIAD1 fusion; lane 12: MBP_Nlinker_-ApxIAD1 fusion

Recombinant expression of the Apx toxins has been reported to result in the formation of insoluble inclusions bodies [32–34]. The presence of extensive regions of hydrophobicity at the N-terminus (including nine amphipathic α-helices), responsible for the formation of cation-selective pores in target cell membranes [35, 36], largely explains this apparent insolubility. Although some glycoproteins are insoluble or membrane bound [37, 38], glycosylation is a soluble phenomenon and we speculated that the formation of inclusions bodies would hinder cytoplasmic glycosylation of our acceptors by NGT. Therefore, three fragments of the ApxIA toxin, termed ‘hydrophobic domain’, ‘activation domain’ and ‘calcium-binding domain’, were constructed as outlined in Fig. 2a. While expression of the hydrophobic domain in *E. coli* BL21 cells resulted in detectable protein, this was mostly confined to the insoluble fraction (Fig. 2b and c), consistent with reported observations. The calcium-binding domain fragment expressed well, was detectable in the soluble fraction and purified by nickel-affinity chromatography (Fig. 2c, lane 7). Expression of the activation domain was not detectable under these conditions (Fig. 2c). In an attempt to solubilise the hydrophobic domain, two N-terminal maltose-binding protein (MBP)-fusions were constructed. However, expression of this fusion protein did not improve solubility of the hydrophobic domain, which also expressed relatively poorly (Fig. 2c, lanes 11 and 12). Consequently, the calcium-binding domain of ApxIA emerged as the most promising candidate for construction of a glycoconjugate vaccine.

### Engineering of N*X*(S/T) glycosylation sequons into ApxIAD3

**Fig. 3:**
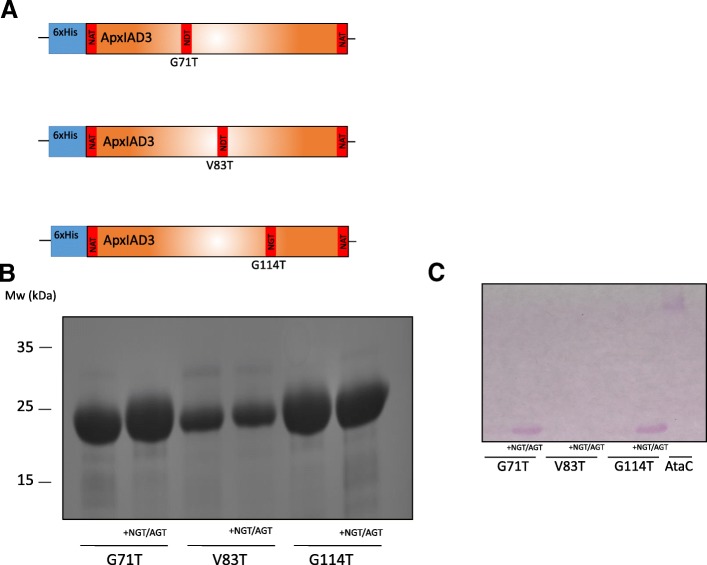
Glycosylation of ApxIAD3 (Ca^2+^-binding domain). **a**- Schematic representation of ApxIAD3. The peptide sequence was engineered to include N- and C-terminal NAT sequons as well as three separate internal modifications. The three ApxIAD3 variants were expressed in *E. coli* with and without chromosomally encoded *ngt* and *agt*. The fragments were purified by Ni-NTA affinity chromatography, resolved by SDS-PAGE and detected by either coomassie stain (**b**) or the presence of glycoprotein detected by PAS staining (**c**)

Although ApxIA encodes 3 N*X*(S/T) sequons that could be targets for NGT, none of these are present within the calcium-binding domain. Therefore, N- and C- terminal NAT sequons were engineered into the amino acid sequence, along with a series of internal modifications to generate appropriate acceptor sites (Fig. 3a). Toxin fragments were expressed in BL21 with and without chromosomally encoded *ngt* and *agt*, purified and resolved by SDS-PAGE. Unlike glycosylation of AtaC with plasmid-borne *ngt* and *agt*, no high molecular weight smearing was observed (Fig. 3b, also see Fig. 5). Toxin fragments migrated at a comparable molecular weight with and without the presence of the transferases. This was consistent with the notion that a shorter glycan had been transferred. Periodic acid-Schiff (PAS) staining was performed to determine whether decoration of these peptides by NGT had been achieved (Fig. 3c). Fragments with the internal modifications G71 T and G114 T were positively stained magenta by the Schiff reagent suggesting that they had been successfully modified. No staining of the V83 T modified fragment was observed, suggesting that glycosylation had not occurred despite the presence of N- and C- terminal NAT sequons. However, this fragment expressed poorly compared with the other fragments, suggesting that that inclusion of a consensus sequon is not the only predetermining factor for glycosylation by NGT and that relative abundance of the target peptide may also be influential.

### Construction of an ApxIIA glycoconjugate

Although *apxIA* is a key virulence determinant for *A. pleuropneumoniae* infections, it is not expressed by all virulent serotypes [4]. Thus, we explored whether this method could be utilised to construct glycoconjugate vaccines for other Apx toxins. ApxI demonstrates greater toxicity than ApxII towards alveolar macrophages and polymorphonuclear neutrophils [39]. However, *apxII* is expressed by all serotypes except 10 and 14 and displays moderate haemolytic and cytotoxic activity. ApxIV is only expressed in vivo and is antigenically distinct from the other Apx toxins [40]. Furthermore, ApxIVA displays a distinct domain architecture from the other toxins, which is reflected in their difference in molecular weight (ApxIV = 202 kDa, ApxI/II = 105 kDa). Due to the observed solubility and glycosylation of the ApxIA Ca^2+^-binding domain, we limited our construction of toxin fragments to the Ca^2+^-regions of ApxIIA and ApxIVA.

**Fig. 4:**
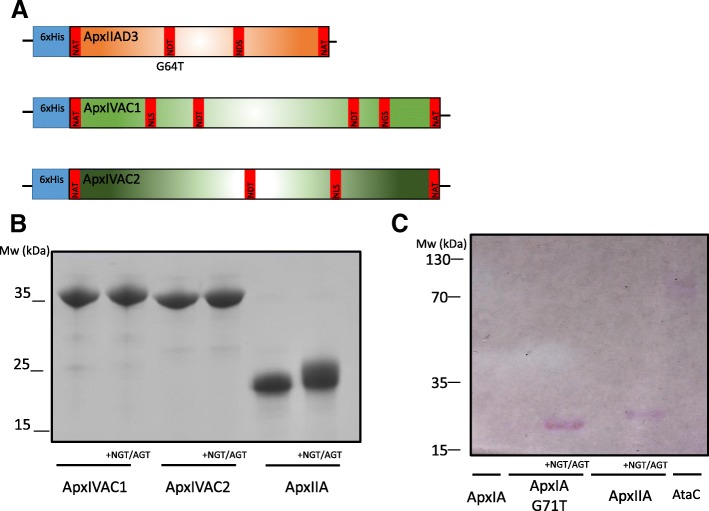
Glycosylation of ApxIVAC1, ApxIVAC2 and ApxIIAD3 (Ca^2+^-binding domain). **a**- Schematic representation of ApxIVAC1, ApxIVAC2 and ApxIAD3. Each peptide was engineered to include N- and C-terminal NAT sequons. ApxIAD3 was further modified to include an internal NDT motif. **b**- The toxin fragments were expressed in *E. coli* with and without chromosomally encoded *ngt* and *agt*. The fragments were purified by Ni-NTA affinity chromatography, resolved by SDS-PAGE and detected by coomassie staining. **c**- The following Ni-NTA purified fragments were resolved by SDS-PAGE and PAS stained: Lane 1: ApxIA (no NX(S/T) sequons); Lane 2: ApxIA (G71 T), no *ngt* and *agt* Lane 3: ApxIA (G71 T) plus chromosomally encoded *ngt* and *agt*; Lane 4: ApxIIA, no *ngt* and *agt*; Lane 5: ApxIIA plus chromosomally encoded *ngt* and *agt*; Lane 6: AtaC, plasmid-encoded *ngt* and *agt*

Unlike ApxIA, the calcium-binding domains of ApxIIA and ApxIVA naturally contain potential sites for NGT glycosylation (1 and 6 sites respectively). For vaccine-fragment construction purposes, the calcium-binding region of ApxIVA was divided into two discrete 35 kDa peptides. ApxIIA was further modified (G64 T) to include an additional internal NDT sequon (Fig. 4a). N- and C-terminal NAT sequons were introduced into all fragments and peptides were expressed in *E. coli* with and without a single chromosomal copy of *ngt* and *agt*. No shift in electrophoretic mobility was observed (Fig. 4b) in the two ApxIVA domains and both were negative by PAS staining (data not shown). A partial shift in electrophoretic mobility was observed for the ApxIIA fragment (Fig. 4b), which also stained positively with Schiff reagent (Fig. 4c) demonstrating that glucosylation had occurred. In agreement with ApxIA glyconconjugate fragment construction (Fig. 3), the presence of recognition sequons within a peptide sequence is not necessarily indicative of glycan modification.

### Glycosylation of Apx toxin fragments by chromosomally encoded *ngt* and *agt* result in a shorter, native-like glycan

**Fig. 5:**
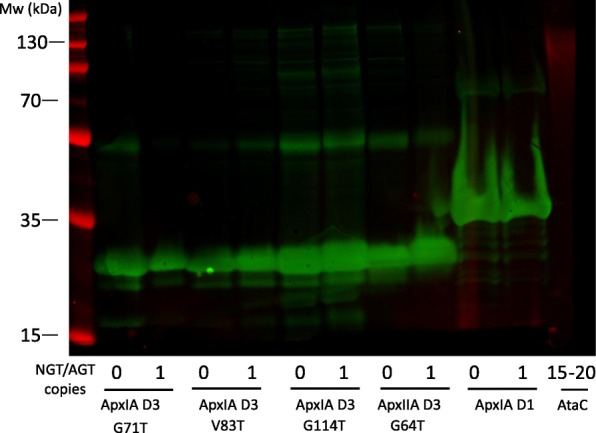
Apx toxin fragments expressed in *E. coli* with and without chromosomally encoded *ngt* and *agt,* were resolved by SDS-PAGE and detected by immunoblot analysis probed with anti-6xHis (green) and anti-dextran (red) antibody. Lanes 2/3: ApxIAD3 (G71 T); lanes 4/5: ApxIAD3 (V83 T); lanes 6/7: ApxIAD3 (G114 T); lanes 8/9: ApxIIAD3 (G64 T); lanes 10/11: ApxIAD1 (whole cell lysate); lane 12: *E. coli* expressing AtaC_1866–2428_ and plasmid encoded *ngt* and *agt*

To assess the polymerisation state of the glucose modification, Apx fragments were probed by immunoblotting with an anti-dextran monoclonal antibody. No reactivity with any of the toxin fragments was observed suggesting that these are not decorated with a glucose polymer (Fig. 5). This observation, taken with the positive PAS stain for fragments ApxIAD3 (G71 T), ApxIAD3 (G114) and ApxIIAD3 (G64 T) (Fig. 3c), suggests that these peptides are modified by NGT and AGT but with no more than 4 glucose units at each site. Given that immunofluorescence studies failed to detect a glucose polymer epitope in *A. pleuropneumoniae* cell lysates [23], we propose that a short glucose repeat unit is representative of a more native-like glycan modification.

### Apx toxin fragments are glycosylated with 1–4 hexose units

**Fig. 6:**
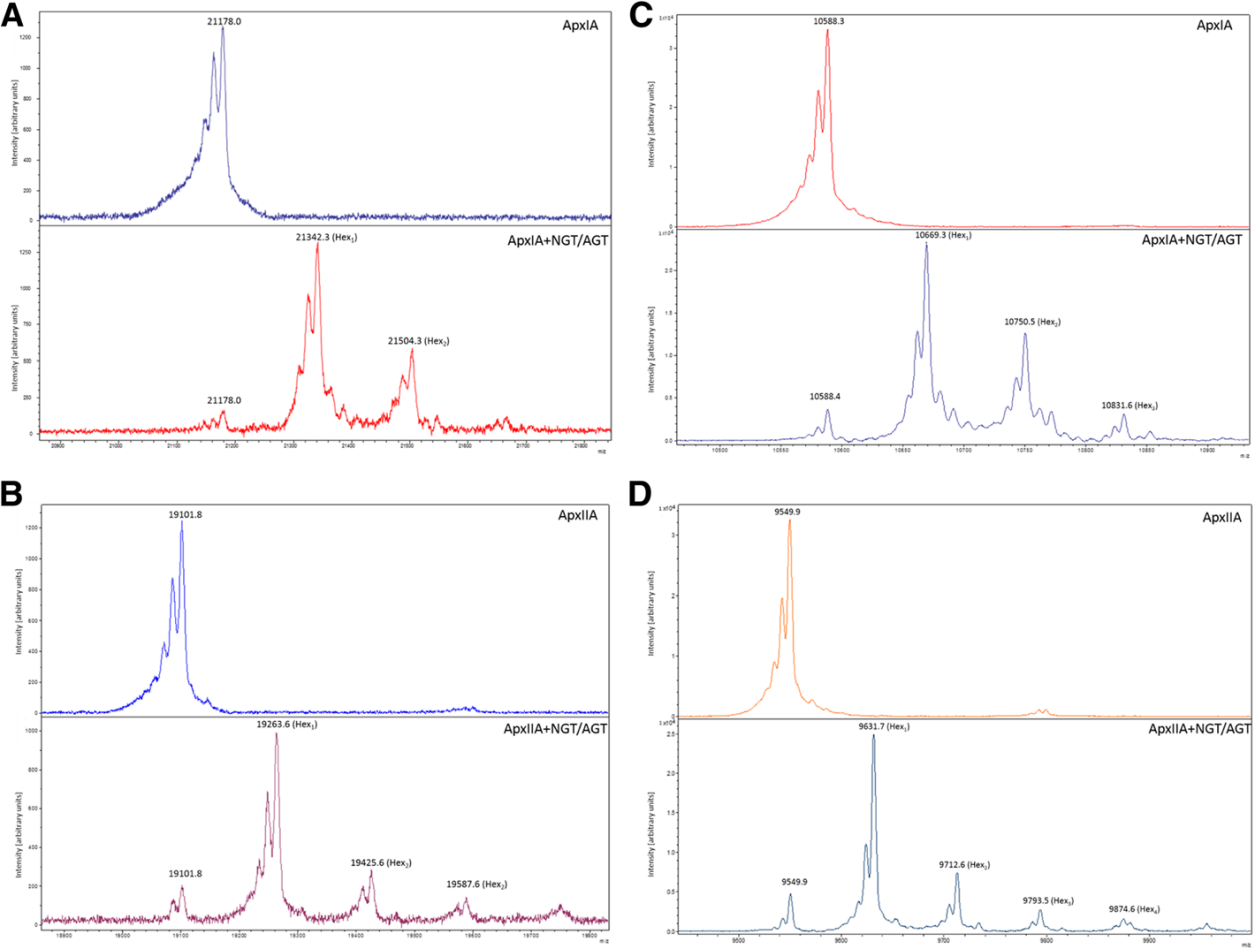
In-tact MALDI-MS analysis of purified ApxIA (**a** and **c**) and ApxIIA (**b** and **d**) expressed from wild type *E. coli* (upper panels) and with *ngt* and *agt* encoded on the chromosome (lower panels). **a** and **b** are peaks corresponding to singly charged ions, **c** and **d** are peaks corresponding to doubly charged ions. The mass shift in the peaks observed in the lower panel in each figure correspond to the addition of hexose units

In order to determine the number of glucose units attached to toxin fragments we performed intact MALDI-MS analysis on ApxIA (G71 T) and ApxIIA (G64 T) purified from cells with and without *ngt* and *agt* encoded on the chromosome. Comparison of the most intense peaks of the glycosylated and unglycosylated forms of ApxIA demonstrates a shift in mass by 162 Da, indicative of a hexose modification (Fig. 6a). Subsequent less intense peaks suggest as many as three hexose units decorate the glycosylated form of ApxIA. Given that ApxIA (G71 T) has three NX(S/T) sequons (hence, three available sites for glycosylation by NGT), it was not immediately clear whether this data suggests occupation of all sites by a single hexose, or by one or two sites with more than one hexoses. Similarly, comparison of the most intense peaks of glycosylated and unglycosylated forms of ApxIIA also indicates a mass shift of 162 Da (Fig. 6b). The ApxIIA fragment contains four potential glycosylation sites and the MALDI spectrum suggests a modification of up to four hexose units. Taken together, these observations indicate that the dominant glycoform of the peptide was modified with a single hexose at a single site. In order to confirm the identity of the observed modification, we performed LC-MS/MS analysis of the glycosylated peptides (Table 1, Additional file 1: Table S1 (full LC-MS/MS output) and annotated spectra in Additional file 2: Figure S1, Additional file 3: Figure S2, Additional file 4: Figure S3). The N-terminal peptide GSNATDISVGK (ApxIA) was identified as being modified with up to 4 hexose units. The peptide GDDEIYGNDTHDILYGDDGNDVIHGGDGNDHLVGGNGNDR (ApxIA) was decorated with 2 hexose units, although the modified asparagine could not conclusively be assigned (Table S1). However, the NDT sequon represents the most plausible location of the hexose addition. No hexose modification was detected on the C-terminal NAT sequon. By contrast, the ApxIIA C-terminal sequon was modified with 1–3 hexoses. Similar to the ApxIA fragment, the N-terminal peptide (GSNATVGNREEK) was modified with 1–4 hexoses. The internal peptide, GDGNDSITDSGGQDK, was modified with 1–2 hexoses. Overall, these data demonstrate that AGT was active when expressed as a single copy encoded on the chromosome. The absence of an extended glucose homopolymer is consistent with previous observations that both glycosylated and unglycosylated peptides resolve at a comparable molecular weight and do not react with an anti-dextran antibody. We propose a shorter glucose polymer may represent a more native-like glycan and thus represents more appropriate modification for a subunit vaccine.

**Table 1:** Identification of glycosylated peptides

Peptide	Tryptic peptide sequence	Glycan modification	Glycan location
ApxIA	GS**N**ATDISVGK	Hex1–4	1xHex (4) [N3(100)]
ApxIA	GDDEIYGN*D*THDILYGDDGNDVIHGGDGNDHLVGG*N*G*N*DR	Hex2	2xHex [N]
ApxIIA	GS**N**ATVGNREEK	Hex1–4	1xHex (4) [N3(100)]
ApxIIA	LAF**N**ATK	Hex1–3	1xHex (3) [N4(100)]
ApxIIA	TGDG**N**DSITDSGGQDKLAF**N**ATK	Hex 1–2, Hex2	1xHex (2) [N5(100)]; 2xHex [N5(100); N20(100)]
ApxIIA	TGDG**N**DSITDSGGQDK	Hex1	1xHex [N5(100)]

## Discussion

In this study, we report the design and construction of *A. pleuropneumoniae* glycoconjugate vaccine candidates. These candidates, based on peptides derived from the ApxIA and ApxIIA toxins coupled with a conserved glycan, have the potential to confer protection against all known virulent serovars of *A. plueropnuemoniae*. Furthermore, we present a convenient mechanism for construction of recombinant glycoproteins by integrating the glycosyltransferases onto the host chromosome. We propose that this results in a more native-like glycan and has the added benefit of reducing metabolic burden of maintaining an additional expression plasmid.

Currently, vaccines against *A. pleuropneumoniae* are based on either inactivated whole cell-bacterins or the Apx toxins. While bacterins offer good protection against the vaccination strain, they offer limited cross-protection against other serotypes [41, 42]. Vaccination with Apx toxins results in reduced morbidity associated with infection but no vaccine to date has been effective in preventing colonisation [42, 43]. Consequently, asymptomatic carriage can increase the risk of infection in non-vaccinated pigs within the herd. Both bacterins and current toxin-based vaccines rely on large-scale growth of live *A. pleuropneumoniae* for production. The use of recombinant *E. coli* offers a safer and more tractable means to generate *A. pleuropnuemoniae* vaccine candidates. Identifying protective antigens that offer cross-serotype protection, reduce disease symptoms and prevent colonisation remains a challenge. However, *E. coli* confers greater flexibility to overcome some of the problems associated with current methods. Recently, the fimbral protein, ApfA, and outer membrane lipoprotein, VacJ, were identified as promising vaccine candidates, due to the fact that they are surface exposed and well-conserved across serotypes [44]. Vaccination with these proteins resulted in increased IgG titre when outer membrane vesicles were used as an adjuvant. However, despite a promising antibody response, this did not translate into effective protection against *A. pleuropneumoniae* infection [45]. Therefore, the toxins remain the most relevant candidates for prevention of disease. We propose that construction of a glycoconjugate that combines a glycan involved in initial cell attachment with toxin fragments may offer protection against colonisation and disease progression.

In this study, we demonstrate a method for construction of a short polymer glycoconjugate, which is more representative of the native glycan epitope. Using *E. coli* as tool for bioengineering of glycoconjugate vaccines has a number of advantages, as well as some challenges, compared with using the original host organism. Previous work has demonstrated that expression of *ngt* and *agt* from a multi-copy plasmid results in a glycan modification not detectable in *A. pleuropneumoniae* itself [20, 22, 23]. Balancing the requirement for high yield of recombinant vaccine without generating non-native by-products was overcome by integrating single copies of the transferases onto the chromosome. Previous observations demonstrate that HMWC1-like transferases demonstrate greater specificity towards their cognate autotransporter adhesins and non-cognate *E. coli* outer membrane proteins [21]. However, they also demonstrate specificity towards sequon-containing peptides that are highly abundant (e.g. DnaK) [21, 46]. We found that chromosomally encoded *ngt* and *agt* and expression of the toxin fragment substrate from a high copy vector resulted in a high yield of soluble vaccine with a shorter glycan (1–4 hexoses) modification.

Recombinant multicomponent subunit toxin-based vaccines have been demonstrated to provide cross protection against a number of *A. pleuropnuemoniae* serotypes [34]. The use of *E. coli* to produce immunogenic peptides obviates the requirement to use virulent strains of *A. pleuropneumoniae* in the vaccine production process. However, a number of studies have highlighted the issue of poor solubility when recombinantly expressing Apx toxins [32–34]. A recent study demonstrated that an ApxI/II/III triple fusion peptide, expressed in *E. coli*, could offer protection in a murine challenge model when delivered in outer membrane vesicles [47]. The peptide portion of each toxin incorporated into the fusion vaccine was remarkably similar to that described in this study i.e. the calcium-binding domain. Crucially, this research demonstrated that the Apx hydrophobic pore-forming domain does not necessarily need to be included in the vaccine composition for it to provide protection and that other toxins domains can constitute immunogenic epitopes. Here, the calcium-binding domains were fused to cytolysin A (ClyA) in order to elicit high antibody titres. Whether the calcium-binding domains alone are sufficient to stimulate a protective immune response warrants further investigation. However, glycosylation of such a fusion by NGT and AGT should be feasible, given that ClyA can be expressed as a soluble peptide. Using soluble fragments is an absolute requirement for glycosylation by cytoplasmic glycosyl transferases but also alleviates challenges associated with purification of peptides from inclusion bodies. Another strategy for generating soluble toxin epitopes is to fuse them to other immunogenic peptides. One study demonstrated that immunisation of mice with the fragment DDEIYGNDGHP fused to *E. coli* heat labile enterotoxin, EtxB, elicited formation of haemolysin neutralising antibodies in mice [9]. This peptide is also part of the calcium-binding domain fragments constructed in this study, although we modified the peptide sequence to DDEIYGNDTHP (ApxIA G71 T) in order to form an NGT acceptor sequon. Taken together, these observations suggest that regions of the toxins other than the pore-forming domains can be used to elicit an immune response, but they are often coupled to other immunogenic peptides.

While a native-like glycan may be more desirable for an *A. pleuropneumoniae* vaccine, dextran-based conjugates have been shown to be useful vaccines against bacteria such as *Helicobacter pylori* [48]. Here, three dextrans with average molecular masses of 5 kDa, 3.5 kDa and 1.5 kDa were fused to tetanus or diphtheria toxoid. These glyconconjugates were immunogenic in rabbits and mice and post-immune sera demonstrated reactivity with α(1–6)-glucan positive strains of *H. pylori* [48]. We present evidence that the length of the glycan can be tailored to generate either a long or a short chain by varying the copy number of the transferases. Furthermore, the *H. influenzae* adhesins have been proposed as potential vaccine candidates due to their conserved nature and contribution to colonisation [26–29]. Although the effect of the glucose modification on adhesin folding and trafficking is well studied [18, 19, 49–51], its interaction with the immune system is currently poorly understood. While these studies have demonstrated the protective properties of antibodies raised against these adhesins, they did not explore whether the glycan itself represents a true epitope. Combining the glycan with a toxin fragment represents a novel approach to generating antigens that may be important in colonisation.

Glycosylation of acceptor sequons was only observed in 3 out of 6 toxin fragment peptides tested, despite all peptides containing terminal NAT sequons. We used LC-MS/MS to demonstrate glycosylation of the following sequons: SNATD (N-terminal, ApxIA), SNATV (N-terminal, ApxIIA) GNDSI (internal, ApxIIA) and FNATK (C-terminal, ApxIIA). Three out of four peptides contained non-polar residues at the − 1 position (the other being Ser). One out of four contained Asp at the + 1 position (the other being Gly). The presence of a Asp residue at the + 1 position was shown to be inhibitory to glycosylation by a recent study [52]. However, our evidence suggests that an Asp at the + 1 position was not an obstacle to glycosylation by NGT, which is consistent the observation that numerous NDT sites within autotransporter adhesins were modified by ApNGT, when expressed in *E. coli* [21, 23]. Asp (negative), Arg (positive), Ile and Val (both non-polar) occupy the + 3 position. By contrast, the sites that were not glucosylated include GNDTI (ApxIA V83 T), NNLSG, GNDTL, GNDTI, INGSY (all ApxIVAC1), GNDTV and INLSE (both ApxIVAC2). Similar to those peptides that were glycosylated, 7 of 8 contain non-polar residues at the − 1 position (the other being Asp). A combination of non-polar and negatively charged residues were observed at the + 1 position (3 non-polar, 4 Asp). Taken together, we can infer no reason in principle why the primary amino acid sequence of these unglycosylated peptides alone should be intrinsically prohibitive to modification by NGT. Therefore, we propose that the absence of glycosylation may be a consequence of poor substrate protein expression. Our data indicated that the vaccine candidates that demonstrated the greatest stability or abundance were the substrates that were glycosylated.

LC-MS/MS analysis demonstrated that toxin fragments were decorated with between 1 and 4 hexoses. The N-terminal NAT sequons were modified with 4 hexoses in both ApxIA and ApxIIA fragments. By contrast, the C-terminal NAT sequon and internal NDS sequon of ApxIIA were decorated with 1–2 or 1–3 hexoses, respectively. The reasons for this heterogeneity are unclear, but suggest that AGT may demonstrate some substrate site specificity. Whether this is dictated by primary amino acid structure warrants further investigation. However, immunisation with heterogeneous glycoforms presents the immune system with a variety of different epitopes, which could be advantageous. In fact, almost all chemically conjugated glyconconjuate vaccines are heterogeneous in regards to polysaccharide length and attachment site [53, 54].

## Conclusions

We describe a method for the assembly of novel glycoconjugate vaccine candidates to protect pigs against the respiratory pathogen, *A. pleuropneumoniae.* By integrating NGT and AGT into the *E. coli* chromosome we were able to generate Apx toxin fragments modified with a glycan that more closely resembles that observed on *A. pleuropnuemoniae* high molecular weight adhesins. While toxins confer the best protection against disease, reducing colonisation limits the risk of disease spread by asymptomatic carriers. Conjugating a toxin fragment with an adhesin-associated glycan offers a novel approach to generating epitopes that may be important for both colonisation and disease.

## Materials and methods

### Bacterial strains and culture conditions

Bacterial strains and plasmids used in this study are listed in Additional file 5: Tables S2 and Additional file 6: Table S3 respectively. *E. coli* strains were cultured in LB broth or agar (Merck, Millipore) at 37 °C and supplemented, when required, with 50 μg ml^− 1^ kanamycin, 100 μg ml^− 1^ ampicillin, μg ml^− 1^ 100 μg ml^− 1^ zeocin, 100 μg ml^− 1^ trimethoprim and diaminopimelic acid (DAP).

### Chromosomal integration of *ngt/agt*

A chromosomal insertion of *ngt* and *agt* was generated using the mini-Tn*5* Km transposon system. Briefly, *ngt, agt* and the lacI repression were amplified from the plasmid pEXT20ngtagt by PCR (Phusion polymerase, New England Biolabs [NEB]) (see Additional file 7: Table S4 for primers used), restriction enzyme digested with NotI (NEB) was and ligated into pUC57, generating plasmid pUC57ngtagt. pUC57ngtagt was digested with SfiI and ligated into plasmid pUTminiTn*5*Km2, generating pUTTn*5*ngtagt, which was transformed into Mu Free Donor (MFD) conjugative strain, a DAP auxotroph [55]. Overnight cultures of MFD and BL21 were subcultured into LB and grown to exponential phase. Cells were sedimented by centrifugation and pellets resuspended in phosphate buffered saline (PBS, Sigma). Cell suspensions were mixed in a 3:1 donor:recipient ratio and were spotted onto antibiotic-free LB agar plates supplemented with DAP, which were grown for 16 h at 37 °C. Cells were scraped from the agar surface using a loop and resuspended in PBS, serial diluted and plated on zeocin selective LB agar plates, without DAP. Colonies were replica plated onto zeocin and ampicillin plates to select for integration into the chromosome and loss of the plasmid backbone (Zeocin resistant, ampicillin sensitive).

### Cloning and synthesis Apx toxin fragments

ApxIA fragments corresponding to the ‘hydrophobic’ domain (D1), ‘activation’ domain (D2) and calcium binding domain (D3) were amplified by PCR from a genomic DNA extraction of *A. pleuropneumoniae* L20 (serotype 5b) [56] using the primers shown in Table S4. PCR products were digested with BamHI and HindIII and ligated into digested pUC19 and pET28a. Plasmids sequences were verified by Sanger sequencing and transformed into *E. coli* BL21. All toxin fragments contained N-terminal hexa His tags for Ni-NTA affinity purification.

MBP constructs were generated using a *malE* gene fragment synthesised by Integrated DNA Technologies (IDT), USA, (see Additional file 8: Figure S4 for sequence). Primer pairs apxIAD1MBP_F/apxIAD1MBP_R and apxIAD1MBP_F/apxIAD1MBP_Nlinker_ R were used to amplify MBP alone and MBP with an asparagine-linker region. PCR products were digested with BamHI and EcoRI and ligated into pET28a. ApxIAD1 was amplified from pUC19apxIAD1 using primers MBPapxIAD1_F2 and apxIAD1_R. The PCR product was digested with SacI and HindIII and ligated into pET28a containing MBP or MBP_Nlinker_.

ApxIAD3 fragments with N- and C-terminal NAT sequons and internal NAT sequon were synthesised as gBlocks by IDT (see Additional file 8: Figure S4 for sequence). Constructs were restriction enzyme digested with PstI and EcoRI and subcloned pUC19. These plasmids served as the template for amplification with apxIAD3NAT primers. PCR products were restriction enzyme digested with BamHI and HindIII ligated into pET28a.

ApxIIAD3, ApxIVAC1 and ApxIVAC2 fragments were synthesised as gBlocks by IDT, codon optimised by expression in *E. coli* (see Additional file 8: Figure S4 for sequences). Constructs were restriction enzyme digested with BamHI and HindIII ligated into pET28a.

### Expression and purification of Apx toxin fragments

Plasmids encoding Apx toxin fragments were transformed into BL21 and BL21::*ngtagt*. Strains were cultured in LB broth, in an orbital shaker, supplemented with appropriate antibiotics to early exponential phase (OD600nm = 0.5) upon which expression was induced by the addition of 1 mM Isopropyl β-D-1-thiogalactopyranoside (IPTG, Sigma). Cultures were incubated for a further 16 h, sedimented by centrifugation and resuspended in 50 mM Tris HCl pH 8, 300 mM NaCl, 10 mM imidazole. Cells were lysed using BeadBug zirconium lysing tubes (Sigma) in a FastPrep homogeniser (MP Biomedicals) and insoluble material was removed by pelleting. Toxin fragments were subsequently purified from cell lysates by Ni-NTA affinity chromatography and eluted in 50 mM Tris HCl pH 8, 300 mM NaCl, 300 mM imidazole.

### Immunoblot analysis

Peptides were resolved by SDS-PAGE and transferred to nitrocellulose membranes using the iBlot 2 dry blotting system (ThermoFisher). Membranes were blocked for 1 h in PBS containing 2% (*w*/*v*) skimmed milk powder. Primary antibodies, Rabbit anti-6xHis antibody (Abcam, UK, used at a dilution of 1:10000) and mouse anti-dextran (MS α-Dextran Clone Dx1, Stem Cell Technologies, Canada, used at a dilution of 1:2000) suspended in PBS containing 2% w/v skimmed milk powder and 0.1% (*v*/v) Tween 20, were incubated with the membrane for 1 h. Membranes were washed three times with PBS and incubated for 45 min with a secondary goat anti-rabbit IgG IRDye680 or goat anti-mouse IgG IRDye800 (LI-COR Biosciences, UK, both at a dilution of 1:10000). Fluorescent signal was detected with the Odyssey LI-COR detection system (LI-COR Biosciences, UK).

### Periodic acid Schiff (PAS) staining

Apx toxin fragments were purified by Ni-NTA affinity chromatography, protein concentration was quantified using Bradford reagent (Sigma) and equal amounts of protein were resolved by SDS-PAGE. Gels were fixed in 50% (v/v) methanol and PAS staining was performed using the Pierce Glycoprotein Staining Kit (ThermoFisher) according to the manufacturer’s instructions.

### LC-MS/MS

Protein solutions were subjected to reduction (DTT) and alkylation (iodoacetamide) prior to overnight digestion with trypsin (Promega, Winsconsin, USA). After digestion, the solutions were pipetted into sample vials and placed in the LC autosampler.

LC-MS/MS experiments were performed using a Dionex Ultimate 3000 RSLC nanoUPLC (Thermo Fisher Scientific Inc., Waltham, MA, USA) system and an Orbitrap Lumos mass spectrometer (Thermo Fisher Scientific Inc., Waltham, MA, USA). Peptides were loaded onto a pre-column (Thermo Scientific PepMap 100 C18, 5 μm particle size, 100A pore size, 300 μm i.d. × 5 mm length) from the Ultimate 3000 auto-sampler with 0.1% formic acid for 3 min at a flow rate of 10 μL/min. After this period, the column valve was switched to allow elution of peptides from the pre-column onto the analytical column. Separation of peptides were separated by C18 reverse-phase chromatography at a flow rate of 300 nL/min and a Thermo Scientific reverse-phase nano Easy-spray column (Thermo Scientific PepMap C18, 2 μm particle size, 100A pore size, 75 μm i.d. × 50 cm length) at a flow rate of 300 nL/min). Peptides were loaded onto a pre-column (Thermo Scientific PepMap 100 C18, 5 μm particle size, 100A pore size, 300 μm i.d. × 5 mm length) from the Ultimate 3000 autosampler with 0.1% formic acid for 3 min at a flow rate of 10 μL/min. After this period, the column valve was switched to allow elution of peptides from the pre-column onto the analytical column. Solvent A was water + 0.1% formic acid and solvent B was 80% acetonitrile, 20% water + 0.1% formic acid. The linear gradient employed was 2–40% B in 30 min. (Total LC run time was 60 mins including high organic wash step and column re-equilibration).

The eluted peptides from C18 column LC eluant were sprayed into the mass spectrometer by means of an Easy-Spray source (Thermo Fisher Scientific Inc.). All *m/z* values of eluting peptide ions were measured in an Orbitrap mass analyzer, set at a resolution of 120,000 and were scanned between *m/z* 380–1500 Da. Data dependent MS/MS scans (3 s cycle time) were employed to automatically isolate and fragment precursor ions and generate fragment ions by higher energy collisional-induced dissociation (HCD) (Normalised Collision Energy (NCE): 38%) in the ion routing multipole. The resolution of the Orbitrap was set to 15,000 for the measurement of fragment ions. Singly charged ions, ions with greater than seven charges and ions with unassigned charge states were excluded from being selected for MS/MS and a dynamic exclusion window of 70 s was employed.

Post-run, the data was processed using Protein Discoverer (version 2.1., Thermo Scientific). Briefly, Orbitrap raw files were searched using SequestHT algorithm against database containing the ApxIA or ApxIIA proteins and common contaminant proteins (such as trypsin, BSA and keratins). Static modification carbamidomethyl on cysteine was applied. Also were considered the dynamic modifications of 1, 2, 3, and 4 hexose units on asparagine (+ 162.1 Da, + 324.1 Da, + 486.2 Da and + 648.2 Da respectively). The peptide and fragment mass tolerances were set to 20 ppm and 0.5 Da, respectively. FDR was estimated with Percolator node and glycosylation site reliability was calculated with ptmRS node. Only high-confidence peptides (FDR < 1%) and glycosylation sites (ptmRS sore > 75%) were accepted in the consensus step. All identifications from common contaminant database were manually removed.

### Intact MALDI-MS

Samples were desalted with C18 Ziptip™ (Millipore) using manufacturers protocol. Each eluent was left to dry on the polished steel MALDI plate and 2 μl of 2,5- Dihydroxyacetophenone matrix (Sigma Aldrich) at 18 mg/ml in ethanol was applied on top. Spectra were acquired using Bruker UltrafleXtreme MALDI mass spectrometer using positive linear mode at up to 50% laser power. Bruker flexControl 3.4 software was utilized for MALDI MS spectra analysis.

## Additional files

Additional file 1: **Table S1.** Full LC-MS/MS output. (XLSX 15 kb)

Additional file 2: **Figure S1.** MS/MS spectrum corresponding to peptide GDDEIYGNDTHDILYGDDGNDVIHGGDGNDHLVGGNGNDR from ApxIA modified with 2 hexose units, showed continuous fragmentation ions, which confirm the peptide identity. However, the modified site could not be conclusively determined. (PDF 142 kb)

Additional file 3: **Figure S2.** MS/MS spectrum corresponding to peptide GSNATDISVGK from ApxIA modified with 4 hexose units, showed continuous fragmentation ions, which confirm the peptide identity. (PDF 125 kb)

Additional file 4: **Figure S3.** MS/MS spectrum corresponding to peptide TGDGNDSITDSGGQDKLAFNATK from ApxIA modified with 1 (GNDSI) and 3 (FNATK) hexose units, showed continuous fragmentation ions, which confirm the peptide identity. (PDF 133 kb)

Additional file 5: **Table S2.** Strains used in this study. (DOCX 15 kb)

Additional file 6: **Table S3.** Plasmids used in this study. (DOCX 16 kb)

Additional file 7: **Table S4.** Oligonucleotides used in this study. (DOCX 13 kb)

Additional file 8: **Figure S4.** DNA sequences of gBlock gene fragments synthesised for this study. (PDF 174 kb)
