# Supplementary figures and images for: Cytoplasmic glycoengineering of Apx toxin fragments in the development of Actinobacillus pleuropneumoniae glycoconjugate vaccines
Source: BMC Vet Res. 2019 Jan 3;15:6. doi: 10.1186/s12917-018-1751-2 (PMC6318927; doi:10.1186/s12917-018-1751-2)

## Supplementary Figure 1

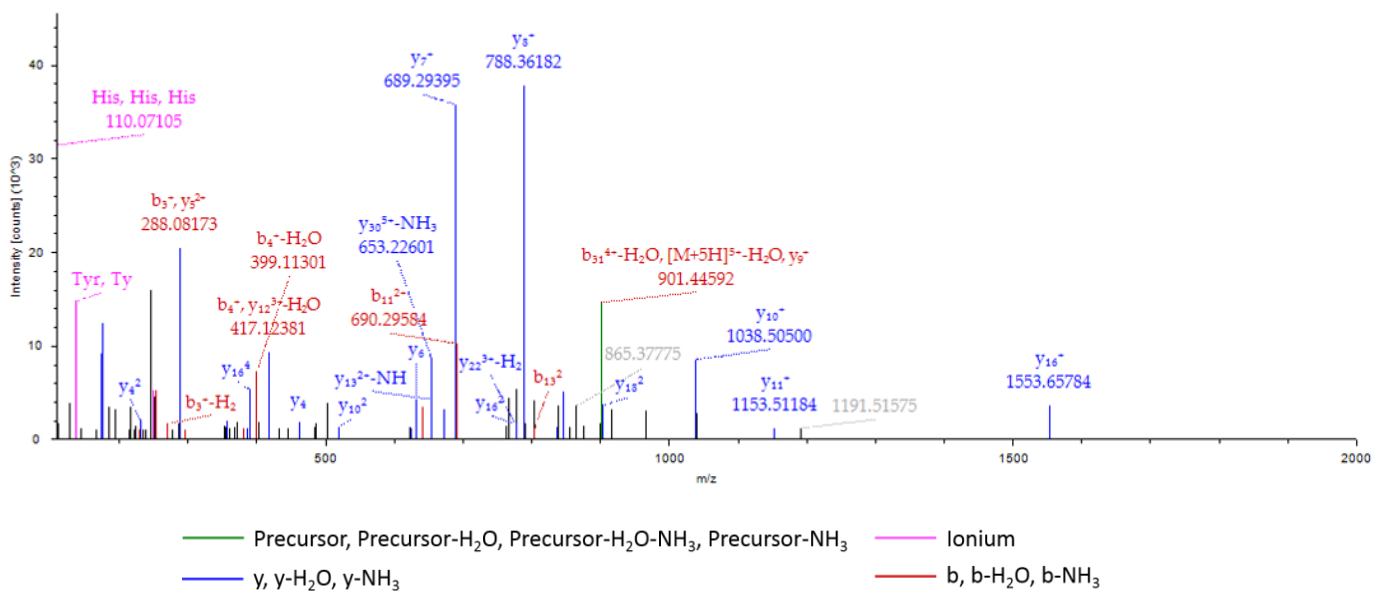

Supplement: Supplementary file 2 — Figure S1. MS/MS spectrum corresponding to peptide GDDEIYGNDTHDILYGDDGNDVIHGGDGNDHLVGGNGNDR from ApxIA modified with 2 hexose units, showed continuous fragmentation ions, which confirm the peptide identity. However, the modified site could not be conclusively determined. (PDF 142 kb) [file 12917_2018_1751_MOESM2_ESM.pdf]

Supplementary Figure 2

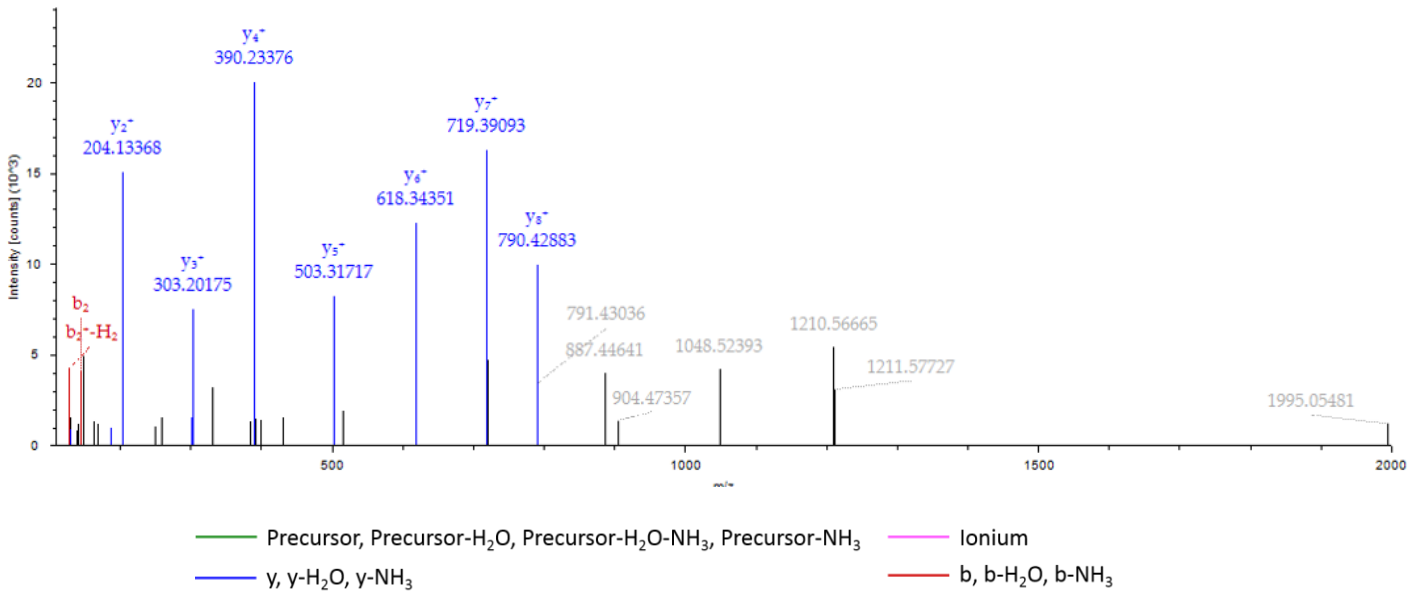

Supplement: Supplementary file 3 — Figure S2. MS/MS spectrum corresponding to peptide GSNATDISVGK from ApxIA modified with 4 hexose units, showed continuous fragmentation ions, which confirm the peptide identity. (PDF 125 kb) [file 12917_2018_1751_MOESM3_ESM.pdf]

# Supplementary Figure 3

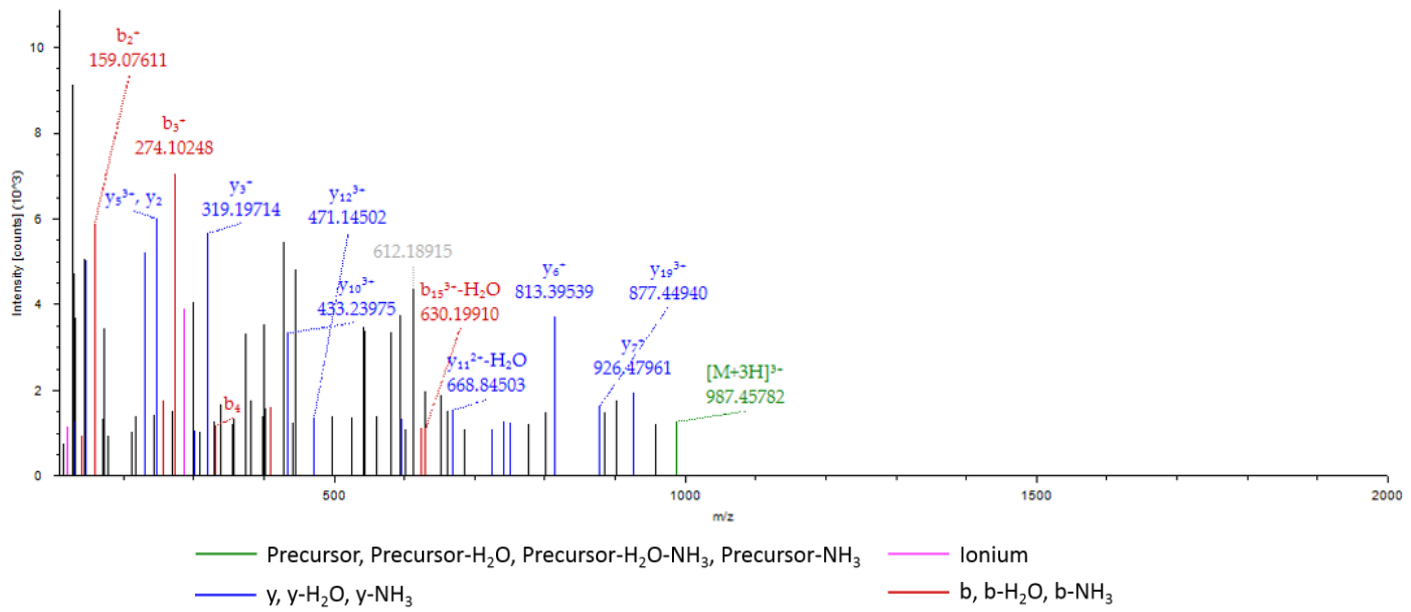

Supplement: Supplementary file 4 — Figure S3. MS/MS spectrum corresponding to peptide TGDGNDSITDSGGQDKLAFNATK from ApxIA modified with 1 (GNDSI) and 3 (FNATK) hexose units, showed continuous fragmentation ions, which confirm the peptide identity. (PDF 133 kb) [file 12917_2018_1751_MOESM4_ESM.pdf]
