# Supplementary material for: Cytoplasmic glycoengineering of Apx toxin fragments in the development of Actinobacillus pleuropneumoniae glycoconjugate vaccines
Source: BMC Vet Res. 2019 Jan 3;15:6. doi: 10.1186/s12917-018-1751-2 (PMC6318927; doi:10.1186/s12917-018-1751-2)
Supplement: Supplementary file 5 — Table S2. Strains used in this study. (DOCX 15 kb) [file 12917_2018_1751_MOESM5_ESM.docx]

**Table S2, Strains used in this study**

| **Strain** | **Genotype** | **Reference** |
| --- | --- | --- |
| *E. coli* 10β | F^–^ *endA1* *deoR*^+^ *recA1* *galE15* *galK16* *nupG* *rpsL* Δ*(lac)X74* φ80*lacZΔM15* *araD139* Δ*(ara,leu)7697* *mcrA* Δ*(mrr-hsdRMS-mcrBC)* Str^R^ λ^–^ | New England Biolabs |
| *E. coli* BL21 (DE3) | *fhuA2* [*lon*] *ompT gal* (λ DE3) [*dcm*] Δ*hsdS* λ DE3 = λ sBamHIo *Δ*EcoRI-B *int*::(*lacI*::P*lacUV5*::T7 gene 1) i21 *Δnin5* T1 phage-resistant version of BL21(DE3) | New England Biolabs |
| *E. coli* BL21::*ngtagt* | BL21::*ngtagt* unknown location of the insertion | This study |
| *A. pleuropneumoniae* L20 |  | Foote *et al.,* 2008^1^ |
| *E. coli* Mu-free donor, MFD*pir* | MG1655 RP4-2-Tc::[ΔMu1::aac(3)IV-ΔaphA-Δnic35-ΔMu2::zeo] ΔdapA::(erm-pir) ΔrecA | Ferrieres *et al.,* 2010^2^ |
