# Supplementary material for: Cytoplasmic glycoengineering of Apx toxin fragments in the development of Actinobacillus pleuropneumoniae glycoconjugate vaccines
Source: BMC Vet Res. 2019 Jan 3;15:6. doi: 10.1186/s12917-018-1751-2 (PMC6318927; doi:10.1186/s12917-018-1751-2)
Supplement: Supplementary file 6 — Table S3. Plasmids used in this study. (DOCX 16 kb) [file 12917_2018_1751_MOESM6_ESM.docx]

**Table S3, Plasmids used in this study**

| **Plasmid** | **Description** | **Source** |
| --- | --- | --- |
| pUC19 | Cloning vector | New England Biolabs |
| pET28a | T7 expression vector | Merck, Millipore |
| pUTminiTn5-Km2 | Conjugative plasmid containing Tn5 transposon | de Lorenzo *et al.,*1990 ^3^ |
| pMLBAD-AtaC | AtaC_1866-2428_ expression vector | Naegeli *et al.,* 2014^4^ |
| pEXT20-ngtagt | *ngt/agt* expression vector | Cuccui *et al.,* 2017^5^ |
| pUC57-ngtagt | Expression vector, including the *lacIQ* expression system | This study |
| pUTminiTn5-ngtagt | *ngt/agt* encoded within Tn5 transposon ends | This study |
| pUC19-apxIAD1 | Maintenance of *apxIA* hydrophobic domain | This study |
| pUC19-apxIAD2 | Maintenance of *apxIA* activation domain | This study |
| pUC19-apxIAD3 | Maintenance of *apxIA* Ca^2+^-binding domain | This study |
| pET28a-apxIAD1 | Expression vector containing *apxIA* hydrophobic domain | This study |
| pET28a-apxIAD2 | Expression vector containing *apxIA* activation domain | This study |
| pET28a-apxIAD3 | Expression vector containing *apxIA* Ca^2+^-binding domain | This study |
| pET28a-apxIAD1MBP | Expression vector containing *malE-apxIA* hydrophobic domain fusion | This study |
| pET28a-apxIAD1MBP_Nlinker_ | Expression vector containing *malE-apxIA* hydrophobic domain fusion with asparagine linker | This study |
| pET28a-apxIAD3(G71T) | Expression vector containing *apxIA* Ca^2+^-binding domain with G71T substitution | This study |
| pET28a-apxIAD3(V83T) | Expression vector containing *apxIA* Ca^2+^-binding domain with V83T substitution | This study |
| pET28a-apxIAD3(G114T) | Expression vector containing *apxIA* Ca^2+^-binding domain with G114 substitution | This study |
| pET28a-apxIIAD3 (G64T) | Expression vector containing *apxIIA* Ca^2+^-binding domain with G64T substitution | This study |
| pET28a-apxIVAC1 | Expression vector containing *apxIVA* Ca^2+^-binding domain 1 | This study |
| pET28a-apxIVAC2 | Expression vector containing *apxIVA* Ca^2+^-binding domain 1 | This study |
