# Supplementary material for: Cytoplasmic glycoengineering of Apx toxin fragments in the development of Actinobacillus pleuropneumoniae glycoconjugate vaccines
Source: BMC Vet Res. 2019 Jan 3;15:6. doi: 10.1186/s12917-018-1751-2 (PMC6318927; doi:10.1186/s12917-018-1751-2)
Supplement: Supplementary file 7 — Table S4. Oligonucleotides used in this study. (DOCX 13 kb) [file 12917_2018_1751_MOESM7_ESM.docx]

**Table S4, Oligonucleotides used in this study**

| **Primer** | **Description** | **Sequence** |
| --- | --- | --- |
| apxIAD1 F | Amplification of *apxIAD1* | TTTTGGATCCAAGCAAGCAGGGCAGAAATT |
| apxIAD1 R | Amplification of *apxIAD1* | TTTTAAGCTTAGCCCCAACACCTGCGGAAG |
| apxIAD1MBP F | Amplification of *apxIAD1* for MBP fusion | TATAGGATCCAAAATCGAAGAAGGTAAACTGGT |
| apxIAD1MBP R | Amplification of *apxIAD1* for MBP fusion | TTTTGAATTCAGTCTGCGCGTCTTTCAGG |
| apxIAD1MBP_Nlinker_ R | Amplification of *apxIAD1* for MBP fusion with asparagine linker | TTTTGAATTCGTTGTTGTTATTGTTATTGTTGTTG |
| MBPapxIAD1 F2 | Amplification of *apxIAD1* for MBP fusion | TTTTGAGCTCAAGCAAGCAGGGCAGAAATT |
| apxIAD2 F | Amplification of *apxIAD2* | TTTTGGATCCATTATTTCAGGTATTTTAGA |
| apxIAD2 R | Amplification of *apxIAD2* | TTTTAAGCTTGTCAAATGTTAAGTAACCTG |
| apxIAD3 F | Amplification of *apxIAD3* | TTTTGGATCCGATATTTCAGTTGGAAAACGC |
| apxIAD3 R | Amplification of *apxIAD3* | TTTTAAGCTTAGTACCATCGCTGCCATACA |
| apxIAD3NAT F | Amplification of *apxIAD3* with engineered NAT sequons | TTTTGGATCCAATGCGACCGATATTTCAGTT |
| apxIAD3NAT F | Amplification of *apxIAD3* with engineered NAT sequons | TTTTAAGCTTGGTCGCATTAGTACCATCGC |
| *ngtagtlaqIQ* NotI F | Amplification of *ngt/agt* from pEXT20- ngtagt | TTTTGCGGCCGCTTCTACGTGTTCCGCTTCC |
| *ngtagtlaqIQ* NotI F | Amplification of *ngt/agt* from pEXT20-ngtagt | TTTTGCGGCCGCATTGCGTTGCGCTCACTGC |
