# Supplementary material for: Cytoplasmic glycoengineering of Apx toxin fragments in the development of Actinobacillus pleuropneumoniae glycoconjugate vaccines
Source: BMC Vet Res. 2019 Jan 3;15:6. doi: 10.1186/s12917-018-1751-2 (PMC6318927; doi:10.1186/s12917-018-1751-2)
Supplement: Supplementary file 8 — Figure S4. DNA sequences of gBlock gene fragments synthesised for this study. (PDF 174 kb) [file 12917_2018_1751_MOESM8_ESM.pdf]

Figure S4, DNA sequences of gBlock gene fragments synthesised for this study

MBP+Nlinker

TTTTGAATTCCCATGGGAAAAATCGAAGAAGGTAAACTGGTAATCTGGATTAACGGCGATAAAGGCTATAAC
GGTCTCGCTGAAGTCGGTAAGAAATTCGAGAAAGATACCGGAATTAAAGTCACCGTTGAGCATCCGGATAAA
CTGGAAGAGAAATTCCCACAGGTTGCGGCAACTGGCGATGGCCCTGACATTATCTTCTGGGCACACGACCGCT
TTGGTGGCTACGCTCAATCTGGCCTGTTGGCTGAAATCACCCCGGACAAAGCGTTCCAGGACAAGCTGTATCC
GTTTACCTGGGATGCCGTACGTTACAACGGCAAGCTGATTGCTTACCCGATCGCTGTTGAAGCGTTATCGCTG
ATTTATAACAAAGATCTGCTGCCGAACCCGCCAAAAACCTGGGAAGAGATCCCGGCGCTGGATAAAGAACTG
AAAGCGAAAGGTAAGAGCGCGCTGATGTTCAACCTGCAAGAACCGTACTTCACCTGGCCGCTGATTGCTGCT
GACGGGGGTTATGCGTTCAAGTATGAAAACGGCAAGTACGACATTAAAGACGTGGGCGTGGATAACGCTGG
CGCGAAAGCGGGTCTGACCTTCCTGGTTGACCTGATTAAAAACAAACACATGAATGCAGACACCGATTACTCC
ATCGCAGAAGCTGCCTTTAATAAAGGCGAAACAGCGATGACCATCAACGGCCCGTGGGCATGGTCCAACATC
GACACCAGCAAAGTGAATTATGGTGTAACGGTACTGCCGACCTTCAAGGGTCAACCATCCAAACCGTTCGTTG
GCGTGCTGAGCGCAGGTATTAACGCCGCCAGTCCGAACAAAGAGCTGGCAAAAGAGTTCCTCGAAAACTATC
TGCTGACTGATGAAGGTCTGGAAGCGGTTAATAAAGACAAACCGCTGGGTGCCGTAGCGCTGAAGTCTTACG
AGGAAGAGTTGGTGAAAGATCCGCGTATTGCCGCCACTATGGAAAACGCCCAGAAAGGTGAAATCATGCCGA
ACATCCCGCAGATGTCCGCTTTCTGGTATGCCGTGCGTACTGCGGTGATCAACGCCGCCAGCGGTCGTCAGAC
TGTCGATGAAGCCCTGAAAGACGCGCAGACTAATTCAAGCTCG
AACAACAACAACAATAACAATAACAACAACCTCGGGATCGAGGGAAGGggccatattgtgtggccggattatgcgaacat
tctgaaagaagtgtttggcggcgcgcgcatggcgtgcgtgaccagcgcgcatatggcgggcgcgaacggcagcattctgaaaaaagcggaag
aaaccagccgcgcgaccatgcataaaccggtgatttttggcgaagattatgtgaccGGATCCTTTT

ApxIA D3 N69 G71T

TTTTGAATTCTTTAAGAAGGAGATATACCATGGGCAGCAGCCATCATCATCATCATCACaatgcgaccgatatttcagt
tggaaaacgcagtgaaaaattagaatatcgtgattatgagttaagcccattcgaacttgggaacggtatcagagctaaagatgaattacattctg
ttgaagaaattatcggtagtaatcgtaaagacaaattctttggtagtcgctttaccgatattttccatggtgcgaaaggcgatgatgaaatctacg
gtaatgacACCcacgatatcttatacggagacgacggtaatgatgtaatccatggcggtgacggtaacgaccatcttgttggtggtaacggaaa
cgaccgattaatcggcggaaaaggtaataatttccttaatggcggtgatggtgacgatgagttgcaggtctttgagggtcaatacaacgtattatt
aggtggtgcgggtaatgacattctgtatggcagcgatggtactaatgcgaccTAACTGCAGTTTT

ApxIA D3 N81 V83T

TTTTGAATTCTTTAAGAAGGAGATATACCATGGGCAGCAGCCATCATCATCATCATCACaatgcgaccgatatttcagt
tggaaaacgcagtgaaaaattagaatatcgtgattatgagttaagcccattcgaacttgggaacggtatcagagctaaagatgaattacattctg
ttgaagaaattatcggtagtaatcgtaaagacaaattctttggtagtcgctttaccgatattttccatggtgcgaaaggcgatgatgaaatctacg
gtaatgacggccacgatatcttatacggagacgacggtaatgatACCatccatggcggtgacggtaacgaccatcttgttggtggtaacggaa
acgaccgattaatcggcggaaaaggtaataatttccttaatggcggtgatggtgacgatgagttgcaggtctttgagggtcaatacaacgtatta
ttaggtggtgcgggtaatgacattctgtatggcagcgatggtactaatgcgaccTAACTGCAGTTTT

ApxIA D3 N112 G114T

TTTTGAATTCTTTAAGAAGGAGATATACCATGGGCAGCAGCCATCATCATCATCATCACaatgcgaccgatatttcagt
tggaaaacgcagtgaaaaattagaatatcgtgattatgagttaagcccattcgaacttgggaacggtatcagagctaaagatgaattacattctg
ttgaagaaattatcggtagtaatcgtaaagacaaattctttggtagtcgctttaccgatattttccatggtgcgaaaggcgatgatgaaatctacg
gtaatgacggccacgatatcttatacggagacgacggtaatgatgtaatccatggcggtgacggtaacgaccatcttgttggtggtaacggaaa
cgaccgattaatcggcggaaaaggtaataatttccttaatggcACCgatggtgacgatgagttgcaggtctttgagggtcaatacaacgtattat
taggtggtgcgggtaatgacattctgtatggcagcgatggtactaatgcgaccTAACTGCAGTTTT

ApxIIAD3 G63T

TTTTGGATCCaatgcgaccgttggtaatcgtgaagaaaaaattgaatatcgtcgtgaagatgatcgttttcatactggttatactgtgacgga
ctcactcaaatcagttgaagagatcattggttcacaatttaatgatattttcaaaggaagccaatttgatgatgtgttccatggtggtaatggtgta
gacactattgatggtaacgatACCgacgatcatttatttggtggcgcaggcgatgatgttatcgatggaggaaacggtaacaatttccttgttgg
aggaaccggtaatgatattatctcgggaggtaaagataatgatatttatgtccataaaacaggcgatggaaatgattctattacagactctggcg
gacaagataaactggcatttaatgcgaccAAGCTTTTTT

ApxIVA Ca^{2+} domain I

TTTTGGATCCAATGCGACCGGCGATGAGAAAAATAACATTTTGCTGGGGTCCCAGAAGGATAATAATTTATCT
GGATCTGCAGGTGACGACCTGCTTATCGGCGGTGAAGGAAACGATACTTTGAAAGGCTCTTACGGTGCTGAT
ACATACTTATTTTCAAAGGGTCACGGCCAGGATGTCATCTACGAATACAGCGACAGCGCGAACTCTAAAAGTG
ATATTGACACTTTAAAGTTTACCGACGTTAACTATGCCGAGGTCAAATTCCGTCGCGTTGGCGACGACCTGATG
TTGTTCGGGTATCACGATACCGACTCCGTTACCGTAAAGAGTTTTTATAACCACGAATATTATCAGTTTGAAAA
GTTGGAGTTCGCAGATCGTTCGATCACCCGTGACGAACTTGGCAAACAGGGAATGGCCTTATTTGGGACCGA
CGGTGACGACGACATCAATGATTGGGGGCGTAACTCAGTCATTGATGCTGGGGCCGGAAATGATACCATTAA
TGGGTCCTATGGAGATGACACGTTGATCGGGGGCACTGGAAACGACATTTTAAAGGGTTCATACGGGGCAGA
CACCTATTTATTTAGCAAAGGTCATGGACAGGACGTAATTTACGAATATAGCGACTCCGCTAATTCTAAACGCG
ACATTGATACATTGAAGTTCACAGATGTAAACTACGCGAATGCGACCAAGCTTTTTT

ApxIVA Ca^{2+} domain II

TTTTGGATCCAATGCGACCTACGGAACGGATGGGAATGACGAAATTAACGACCACGCCGATTGGGATTCAAT
CCTTGAAGGAGGTAAGGGGAATGACATTTTACGCGGGAGCTATGGTGCGGATACCTACATTTTCTCTAAGGG
TCATGGGCAGGATGTTATTTACGAGTATTCTGACAGCGCCAATTCTAAGCGTGATATCGATACATTAAAATTCA
CCGATGTGAATTATGCTGAAGTAAAGTTTCGCCGTGTCGATAACGATTTGATGTTATTTGGATATCACGATACG
GATTCTGTGACCATTAAAAGTTTCTATAACCATGTGGATTATCAGTTTGACAAACTGGAGTTCGCTGATCGCTC
AATCACCCGCGATGAACTTGGGAAGCAGGGTATGGCGTTGTTCGGGACTGATGGTGATGATAACATTAACGA
CTGGGGGCGTAATTCTGTCATTGATGCTGGGGCCGGTAATGACACAGTGAACGGCGGCAACGGCGATGACAC
GTTAATCGGAGGTAAGGGTAACGACATCCTGCGCGGTGGCTATGGTGCAGATACATATATCTTTTCAAAAGG
GCATGGCCAAGATATTGTGTACGAAGACACGAACAATGACAATCGCGCACGCGACATTGATACGTTGAAATTT
ACAGACATTAACCTGTCCGAACTTTGGTTCAGCCGTGAAAACAATGACTTGATCATTAAGTCTTTATTGTCGGA
AGACAAAAATGCGACCAAGCTTTTTT
